# Report of Hygiene, U. S. Army

**Published:** 1875-07

**Authors:** 


### Bibliographical.

---

A REPORT OF THE HYGIENE of the United States Army, with Descriptions of Military Posts. Circular No. 8. Surgeon General's Office, Washington; 1875; quarto; paper; pp. 567.

---

MEDICAL ADDRESSES. By Benjamin Eddy Cotting, A.M., M.D., Harvard, President Massachusetts Medical Society, etc. Boston: David Clapp & Son: 1875; 12mo.; paper; pp. 123.

This pamphlet consists of three separate essays, viz: Nature in Disease; Disease, a part of the plan of creation: and My First Question as a medical student—its solution a sure basis for Rational Therapeutics. The writer treats his subjects in a similar vein with Nature and Art in Disease, by Sir John Forbes. He deprecates the poly-pharmacy of the present day. He would study the natural history of disease. He would ascertain rigidly the *real place of medicines* in the alleviation and cure of disease, and restrict their use within proper limits. The pamphlet furnishes pleasant and useful reading, and may well form the basis of deep and profitable reflection to the medical men of our times.

